# Diagnostic value of abdominal sonography in confirmed COVID-19 intensive care patients

**DOI:** 10.1186/s43055-020-00317-9

**Published:** 2020-09-29

**Authors:** Mohsen Ahmed Abdelmohsen, Buthaina M. Alkandari, Vikash K. Gupta, Ahmed Adel ElBeheiry

**Affiliations:** 1 Department of Radiodiagnosis and Intervention, Faculty of Medicine, University of Alexandria, 10 Shamplion Street, Elazareeta, Alexandria, Egypt; 2 Medical Imaging Department, Ministry of Health, Jaber Al-Ahmad Hospital, Khalid Ben Abdulaziz Street, South Surra, Kuwait City, Kuwait; 3 FFR-RCSI, Dublin, Ireland; 4 University of Rajasthan, Jaipur, India

**Keywords:** COVID-19, Abdominal imaging studies, Hepatomegaly, Cholestasis, Coagulopathy

## Abstract

**Background:**

In December 2019, a large outbreak of a novel coronavirus infection occurred in Wuhan, China. The pneumonic disease caused by this virus is called coronavirus disease 2019 (COVID-19) by the World Health Organization (WHO).

As case numbers have increased worldwide, gastro-intestinal symptoms like diarrhea, constipation, abdominal pain, and vomiting have been increased, these symptoms associated with positive laboratory results including abnormal liver function tests, renal function tests, and D-Dimer levels.

Although there are multiple articles evaluated the imaging findings in HRCT of COVID-19 patients that helped in understanding the disease course and potential complications in the chest, yet there are—to our knowledge—limited data about the abdominal imaging findings of the course and potential abdominal complications of COVID-19 notably in the intensive care units (ICU).

**Results:**

Forty-one sonographic examinations were done for 30 confirmed COVID-intensive care patients presented with abdominal symptoms.

Of the 30 patients, 26 were males (86.66%), and 4 were females (13.3%), the average age of the patients was 57.7 years old.

The most common sonographic observation was hepatomegaly (*n*, 23/41, 56%) and biliary system disease (*n*, 17/41, 41.4%); the imaging findings were correlated with the clinical and laboratory data. CT examination when indicated (in our study to assess hematomas for active extravasation and to assess bowel obstruction and its level).

**Conclusion:**

Abdominal sonographic imaging was often performed for inpatients with COVID-19. Hepatobiliary dysfunction as well as nephropathy was the most common imaging findings.

## Background

In December 2019, a large outbreak of a novel coronavirus infection occurred in Wuhan, China. The novel coronavirus was named severe acute respiratory syndrome coronavirus 2 (SARS-CoV-2) by the International Committee on Taxonomy of Viruses [1–3]. The pneumonic disease caused by this virus is called coronavirus disease 2019 (COVID-19) by the World Health Organization (WHO), on January 30, 2020, the WHO declared a global public health emergency against the outbreak of COVID-19. The WHO recognized the coronavirus disease 2019 (COVID-19) as a worldwide pandemic on March 11, 2020 [1, 2].

The classical symptoms of COVID-19 are dry cough and fever and the diagnosis is confirmed by real-time reverse transcription-polymerase chain reaction (RT-PCR) testing for SARS-Cov-2 nucleic acid. Early radiological studies focused on chest imaging with classical high-resolution CT (HRCT) findings of peripheral patches of ground-glass densities with or without consolidations with bilateral basal predominance, organizing pneumonia pattern, crazy paving, mild bronchiectasis, and vascular engorgement may be encountered as well [4, 5].

As case numbers have increased worldwide, gastro-intestinal symptoms like diarrhea, constipation, abdominal pain, and vomiting have been increased; these symptoms are associated with positive laboratory results including abnormal liver function tests, renal function tests, and D-Dimer levels [6, 7].

Although there are multiple articles evaluated the imaging findings in HRCT of COVID-19 patients that helped in understanding the disease course and potential complications in the chest, yet there are—to our knowledge—limited data about the abdominal imaging findings of the course and potential abdominal complications of COVID-19 [8].

## Aim of the work

This retrospective study was conducted aiming to characterize the sonographic abdominal imaging findings in COVID-19 intensive care patients and to highlight the potential abdominal complications to help in understanding the pathogenesis of the disease in the abdomen.

## Methods

This is a retrospective study analysis of 30 intensive care patients with confirmed COVID-19 infection and presented with abdominal signs and symptoms and/or abnormal visceral laboratory tests.

Each patient was subjected to the following:

I- *Thorough history taking* including the following:
  Recent travel history especially to known epidemic disease territories
  Recent contact with confirmed COVID-19 patient
  Suspicious chest symptoms: fever, dry cough, tremors
  History of underlying medical condition: DM, HTN
  Abdominal signs and symptoms: nausea, vomiting, pain, distention, diarrhea, and constipation
II- *Clinical examination*: Complete clinical examination was done for each patient
III- *Laboratory investigations*

A For confirmation of COVID-19
  Nasopharyngeal and oropharyngeal swab with real-time reverse transcriptase-polymerase chain reaction (RT-PCR) test
  CBC
B Tailored for the clinical scenario
  Liver function tests: especially serum bilirubin, alkaline phosphatase level (ALP)
  Renal function tests
  D-dimer test
  Coagulation profile: INR, partial thromboplastin time, platelet count

IV- *Radiological investigations*
  Ultrasonography: 41 abdominopelvic sonographic examinations were done using a portable GE machine to 30 intensive care patients, due to high human to human transmission incidence, portable ultrasound machine (GE healthcare Voluson E6 BT17 ultrasound machine) was used (to limit transport of the patients to the radiology department).
  MDCT: 3 CT examinations done for 3 patients**,** chest assessment was included in the study, the initial CT protocol was non-contrast scan and post-contrast scan in the venous phase, but this was tailored according to the clinical situation, scans in the arterial phase and delayed phase in the clinical context of abdominal pain and decrease hemoglobin level to investigate possible bleeders and these arterial and delayed scans were added in two patients with suspected spontaneous bleeding. Scans in the arterial and venous phases were added in one patient with suspected bowel obstruction and suspected ischemic bowel etiology. Reconstructions of the volume at 0.625 mm to 1.25 slice thickness, examinations were performed on Siemens Somatom Definition Edge 128 slice helical CT scanner.

Picture archiving and communication system viewer was used (GE PACS).

V- *Data analysis and interpretations*

To decrease the subjective judgment and to reach objective assessment, the following definitions were respected: Gall bladder/bowel mural thickening (when measures more than 3 mm in diameter), gall bladder distention (when the gall bladder measures 4 cm in transverse diameter), acute cholecystitis (positive sonographic murphy’s sign, thickened edematous wall pericholecystic collection, pericholecystic hyperemia).

Hepatomegaly (the liver right lobe measures more than 16 cm in midclavicular line)

VI- *Infection control precautions*

- Using portable machine to limit transport of the patients to the radiology department
- The transported patients to the department for CT examinations must wear a mask
- Droplet type precautions are used. All the attending medical staff should wear personal protective equipment (PPE) including: N95 mask, eye protective devices (googles/face shield), gowns, and gloves
- The machine is covered during the examination
- Proper cleaning following the examination was done

### Inclusion criteria

- Confirmed intensive care patients with COVID-19 infection by positive PCR test for the causative agent (SARS-COV-2 virus) who presented with abdominal signs and symptoms with positive abnormal laboratory studies

### Exclusion criteria

- Confirmed COVID-19 infection by positive PCR test for the causative agent (SARS-COV-2 virus) without abdominal manifestations
- Confirmed COVID-19 infection by positive PCR test for the causative agent (SARS-COV-2 virus) with a history of prior chronic disease, e.g., chronic calcular cholecystitis, prior renal, or hepatic disease

### Ethics approval and consent to participate

Approval for this study was obtained from the Research Ethics Committee of our medical institute. All study procedures were carried out in accordance with the Declaration of Helsinki regarding research involving human subjects. Written consent was waived.

## Results

In this retrospective study, 41 abdominal imaging studies were done for 30 intensive care confirmed COVID-19 patients presented with abdominal symptoms.

Of the 30 patients, 26 were males (86.6%, and 4 were females (13.33%), the age range from 91 to 21 years old, with mean age was 57.7 years old. The patients were distributed in Table 1 according to the age, the largest age group was (more than 50 to 60 years group ) included 8 patients (26.6%) (Table 1).

**Table 1:** Distribution of the studied patients according to demographic data

	Number of the patients (***n*** = 30)	Percentage
**Sex**		
Females	4	13.3
Males	26	86.6
**Age**		
More than 80-90	2	6.6
More than 70-80	**3**	10
More than 60-70	**4**	13.3
More than 50-60	8	26.6
More than 40-50	7	23.2
More than 30-40	4	13.3
More than 20-30	2	6.6

**Fig. 1:**
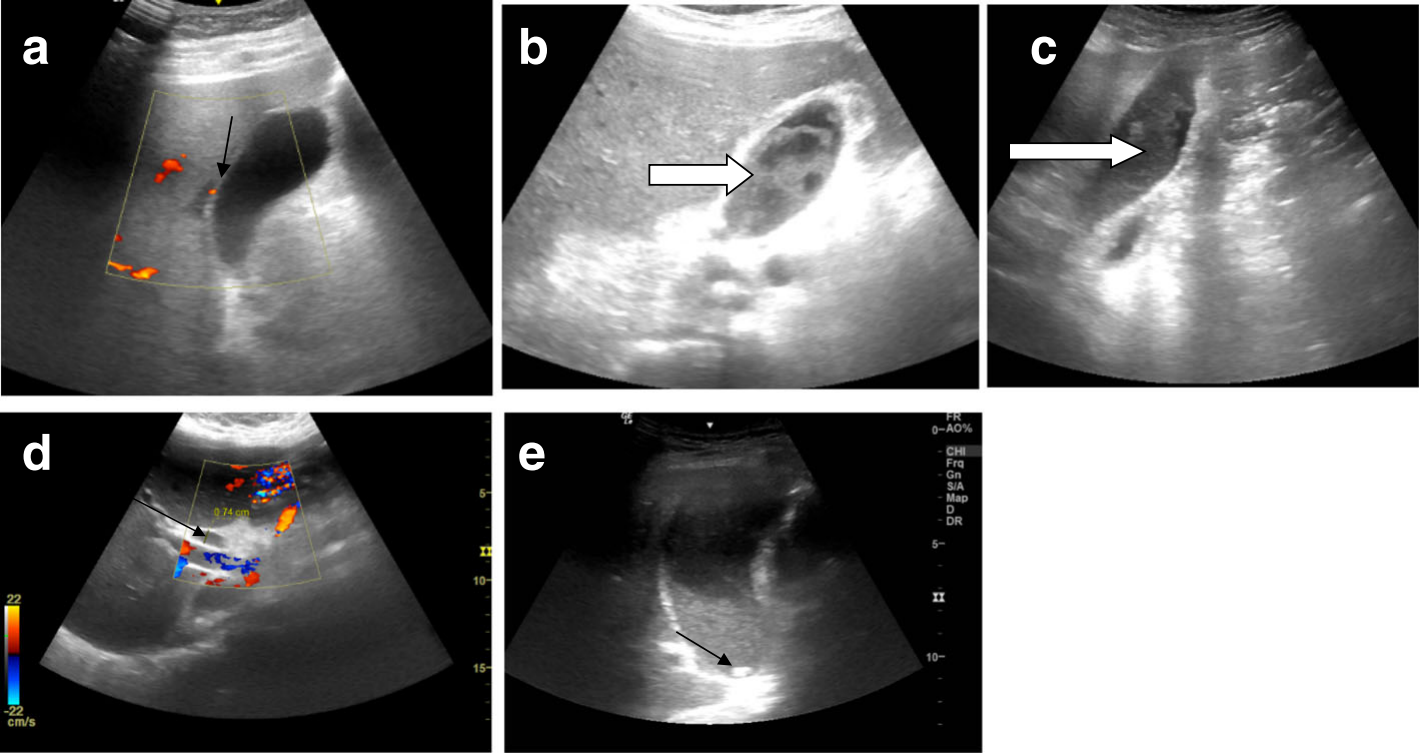
Biliary system disease in five different COVID-19 intensive care patients: (**a**) the gall bladder shows thickened edematous wall with subtle mural hyperemia (arrow) (**b**) and (**c**) marked intraluminal biliary mud (arrows) sequel to cholestasis (**d**) ectatic CBD (7.4 mm) (black arrow) with no definite obstructive lesions probably due to cholestasis (**e**) distended gall bladder with mud, a subcentimeter gall bladder stone is noted (black arrow)

**Fig. 2:**
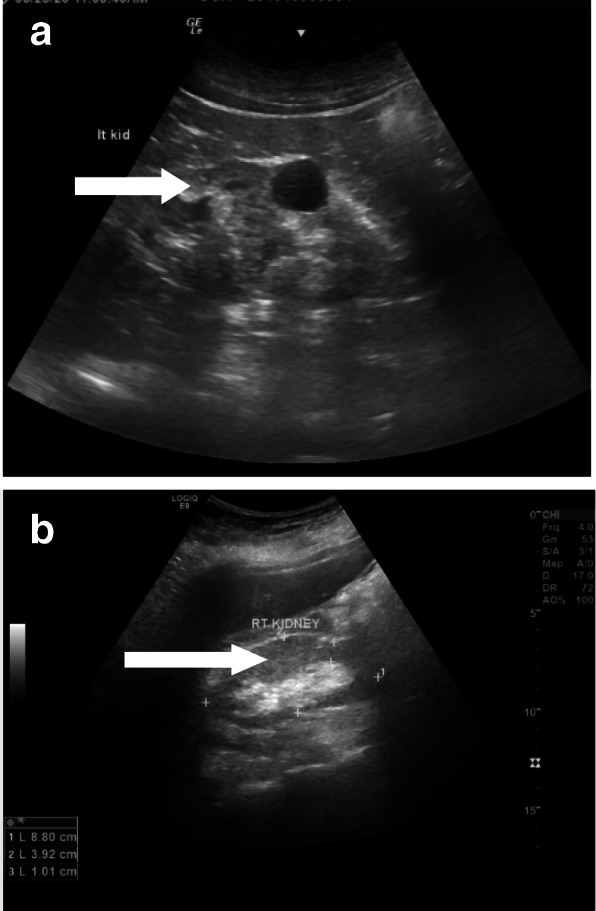
(**a** and **b**) Nephropathy in COVID19 intensive care patient with increased parenchymal echogenicity (open white arrows in **a** and **b**) still with parenchymal thickness and renal measurements within normal range, a midzonal simple cortical cyst is noted in the left kidney (arrow in **a**)

The most frequent indication for sonographic examinations was increase liver function tests especially serum bilirubin (*n*, 21/41; 51.2%) (Fig. 1), followed by increase renal function tests (Fig. 2) (*n*, 6/41;14.6%) (Table 2).

**Table 2:** Distribution of the imaging studies done for the patients sample according to the indications for the imaging studies (NB: 11 follow-up sonographic studies were done for 8 patients)

Indications	ICU patients	Remarkable examinations	Unremarkable	Total number of examinations (***n*** = 41)
**U/S examination**				
Increase liver function tests	13	18	3	21 (51.2%)
Abdominal pain	2	3		3 (7.3%)
Abdominal distention	3	3		3 (7.3%)
Suspected sepsis	2	2		2 (4.8%)
Increase renal function tests	5	4	2	6 (14.6%)
Palpable abdominal swelling	1	1		1 (2.4%)
Decrease hemoglobin	4	3	2	5 (12.1%)

Forty-one sonographic studies were done for 30 intensive care COVID-19 patients, 34 examination were positive for relevant findings (*n*, 34/41; 82.9%), and 7 sonographic studies were unremarkable (n, 7/41; 17.0%) (Table 2).

The most frequent sonographic finding was hepatomegaly (23/41, 56.09%), most of the cases showed increase liver function tests with bright echo pattern of the hepatic parenchyma, biliary system disease was the second most frequent observation (17/41; 41.47%) including gall bladder distention, biliary mud, wall thickening, and dilated common bile duct (Fig. 1).

Nephropathy was the 3rd most common (*n*, 7/41; 17%); the most evident sonographic findings were increase renal parenchymal echogenicity bilaterally as well as parenchymal thinning (Fig. 2) (Table 3).

**Table 3:** Sonographic imaging findings in the studied patient sample (NB: more than one finding in one examination was noted)

Imaging findings	Examinations for ICU patients	Percentage (***n*** = 41)
**Biliary system disease**	**17**	**41.4%**
Acute calcular cholecystitis	10	
Acute calcular cholecystitis	1	
GB thickened wall with intraluminal mud	4	
Prominent CBD with no obstructive lesions	2	
**Hepatomegaly**	23	**56.09%**
**Vasculopathy**		
Spontaneous bleeding	2	4 (9.7%)
Vascular thrombosis	2	
**Nephropathy**	7	17%
Mild free ascetic fluid	5	12.1%

**Fig. 3:**
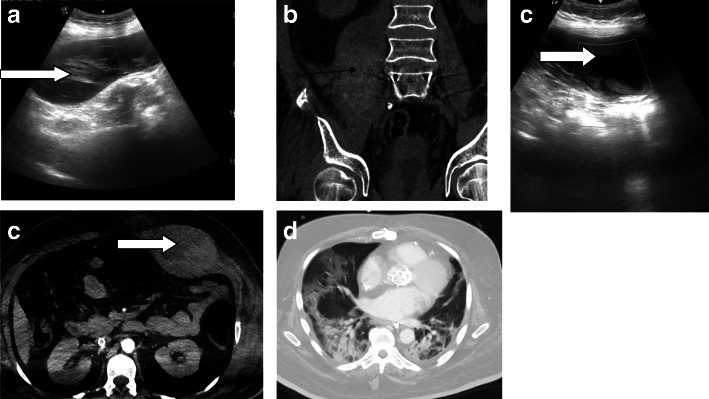
Two different COVID19 patients with spontaneous hematomas (**a**) sonographic assessment of the right psoas spontaneous hematoma (black arrow in **a** and **b**) in COVID-19 patient with corresponding (**b**) non-contrast CT scan (**c**) left rectus sheath hematoma (white open arrow in **c** and **d**) with (**d**) corresponding CT image in the arterial phase with no contrast extravasation (**e**) basal chest scans in the second patient showing bilateral basal patches of consolidation of COVID-19 pneumonia

**Fig. 4:**
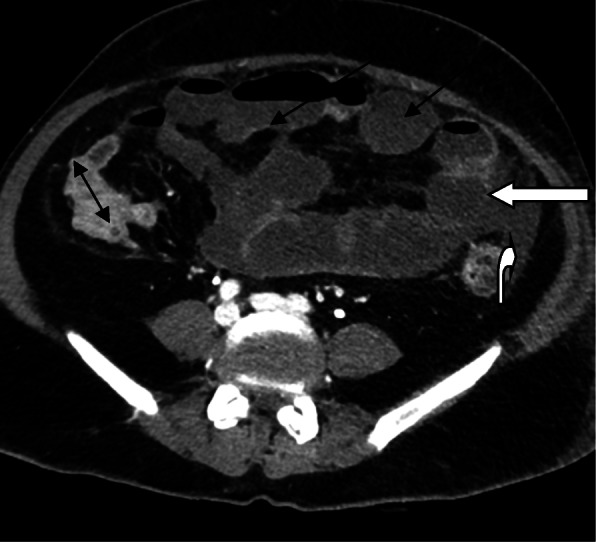
Axial post-contrast CT abdomen done for ICU patient with abdominal distention with limited sonographic assessment with distended small bowel loops (long black arrows), collapsed large bowel loops (double-headed black arrow), there is small bowel segmental mural poor enhancement is noted (straight white open arrow) with related mild collection (curved white open arrow)

Three patients were subjected to CT examinations, the indications for CT examination included to exclude active bleeding in two patients and to assess intestinal obstruction in one patient. Two patients subjected to sonographic studies followed by CT studies for more characterization and to exclude possible active extravasation (one patient with left rectus hematoma and another patient with right-sided retroperitoneal hematoma) (Fig. 3), one patient with abdominal distention with limited sonographic assessment, and CT examination shows small bowel obstruction mostly of ischemic etiology (Fig. 4).

## Discussion

To our knowledge, there is a paucity of literature on sonographic abdominal imaging features of COVID-19 patients, by continuously increasing in number of the confirmed COVID-19 patients all over the world (exceeding 9,700,000 patients at the time of writing), there are increasing frequency of GIT symptoms and ICU admission with the limitation in possible CT examinations for isolated and mechanically ventilated patients, the most common indications for sonographic studies in the ICU were elevated liver function tests (*n*, 21/41; 51.2%), this matches with the COVID-19 frequent abdominal imaging study indications in Bhayana R et al. study [9], this study was done on both ICU and non-ICU patients with more frequent indications in their study like abdominal pain [9].

Up to date, the most acceptable pathogenesis of COVID-19 infection is that SARS-COV2 virus can gain access to the respiratory alveolar epithelial cells by binding to angiotensin-converting enzyme 2 (ACE2) receptors [10], leading to cytokine-mediated immune response and inflammation, imaging findings, and disease course are further affected by severity of the cytokine-mediated inflammation (e.g., cytokine storm syndrome). Circulating cytokines can impact multiple other organ systems, causing secondary diseases and complications [11].

ACE2 receptors are also found in the brain, arterial, and venous vascular endothelial cells, kidney, liver (hepatocytes and cholangiocytes), GIT, and gall bladder epithelial cells. This offers a chance for direct viral invasion of theses organs with variable clinical presentations and imaging spectrum [11, 12].

In our study, male to female ratio was 6.5:1, while in Bhayana et al.’s study [9], male to female ratio was 1.4:1, with similarity in increase male to female ratio in both studies.

Hepatomegaly was the most common findings in sonographic examinations with bright echo patterns reflecting diffuse hepatic parenchymal disease (*n*, 23/41; 56.1%) followed by biliary system disease (*n*, 17/41; 41.4%). Hepatic dysfunction could be noted in about 50% of COVID-19 patients [13].

Ji et al. [14] demonstrated that COVID19 patients with high body mass index and non-alcoholic fatty liver disease (NAFLD) have a greater risk for both progressions in liver damage and COVID-19, in similarity to our study, these patients show increase liver function tests with hepatomegaly. Progression in liver damage related to NAFLD (and potentially other chronic liver disorders) will complicate COVID-19 presentation, clinical course, and the role of imaging, with the potential for hepatic failure, hepatic encephalopathy, and gastrointestinal bleeding [14].

Sonographic findings of biliary disease were noted in 41.4% in our study sample, biliary system disease was noted in 54% in Bhayana et al.’s study [9] spectrum of gall bladder pathology was noted in our study including luminal mud, stone, mural thickening, signs of inflammation with edematous wall, mural hyperemia, and thin rim pericholecystic fluid could be noted, this spectrum of findings is similar to Bhayan et al.’s study with their population sample in their study including ICU and non-ICU patients. SARS-COV2 can bind to the gall bladder epithelial cells leading to mucosal inflammation and this can explain the sonographic observations [14].

SARS-CoV-2 has a direct inflammatory effect on vascular endothelium [15]. Further, systemic coagulopathy is common in critically ill patients with COVID-19 [16, 17]. Tang et al. [16] found that next to an increased risk of thrombosis, patients seem to have an increased risk of bleeding as well, due to imbalances in platelet production and disruption, and disorders of the coagulation system. Coagulopathy sequel in our study was noted in four examinations (9.7%) including spontaneous hematomas noted in two examinations as well as vascular thrombotic sequel noted in two examinations. Major bleeding sequel to COVID-19 was noted in two patients only in Conti C et al.’s published study [17].

Nephropathy was the 3rd most common sonographic observation (*n*, 7/41; 17%) in our study, associated increase in renal function tests was noted. Few studies focused mainly on renal histopathology in COVID-19 patients were published [18, 19]. Further dedicated imaging studies are needed for more clarification.

The authors recommend dealing with COVID-19 as a systemic disease possibly due to possible direct viral cytopathic effect on the ACE-2 receptors rich organs and/or harmful systemic immune-mediated response to SARS-COV 2 infection.

The limitation of this study was that of a single-center retrospective study, which limits its generalizability. Pathologic correlation was not available for many patients with imaging abnormalities.

## Conclusions

Dedicated sonographic abdominal imaging was often performed for COVID-19 intensive care patients with clinicolaboratory abnormal findings with subsequent improvement of the management plan. Hepatobiliary dysfunction, nephropathy as well as coagulopathy sequel in the abdomen were the most common imaging findings. COVID-19 should be considered as a systemic disease.

## Data Availability

The data sets used and/or analyzed during the current study are available from the corresponding author on reasonable request.

## Abbreviations

ICU: Intensive care unit
CT: Computerized tomography
US: Ultrasonography
COVID-19: Coronavirus disease 19
SARA-COV2: Severe acute respiratory syndrome coronavirus
